# Haplotypes at *LBX1* Have Distinct Inheritance Patterns with Opposite Effects in Adolescent Idiopathic Scoliosis

**DOI:** 10.1371/journal.pone.0117708

**Published:** 2015-02-12

**Authors:** Rakesh Chettier, Lesa Nelson, James W. Ogilvie, Hans M. Albertsen, Kenneth Ward

**Affiliations:** 1 Affiliated Genetics, Inc., Salt Lake City, Utah, 84109, United States of America; 2 Lucina Foundation, Salt Lake City, Utah, 84109, United States of America; 3 Juneau Biosciences, LLC., Salt Lake City, Utah, 84109, United States of America; University of Texas, UNITED STATES

## Abstract

Adolescent idiopathic scoliosis (AIS) is a clinically significant disorder with high heritability that affects 2–4% of the population. Genome-wide association studies have identified *LBX1* as a strong susceptibility locus for AIS in Asian and Caucasian populations. Here we further dissect the genetic association with AIS in a Caucasian population. To identify genetic markers associated with AIS we employed a genome-wide association study (GWAS) design comparing 620 female Caucasian patients who developed idiopathic scoliosis during adolescence with 1,287 ethnically matched females who had normal spinal curves by skeletal maturity. The genomic region around *LBX1* was imputed and haplotypes investigated for genetic signals under different inheritance models. The strongest signal was identified upstream of *LBX1* (rs11190878, P_trend_ = 4.18×10^-9^, OR = 0.63[0.54–0.74]). None of the remaining SNPs pass the genome-wide significance threshold. We found rs11190870, downstream of *LBX1* and previously associated with AIS in Asian populations, to be in modest linkage disequilibrium (LD) with rs11190878 (r^2^ = 0.40, D' = 0.81). Haplotype analysis shows that rs11190870 and rs11190878 track a single risk factor that resides on the ancestral haplotype and is shared across ethnic groups. We identify six haplotypes at the *LBX1* locus including two strongly associated haplotypes; a recessive risk haplotype (TTA, Control_freq_ = 0.52, P = 1.25×10^-9^, OR = 1.56), and a co-dominant protective haplotype (CCG, Control_freq_ = 0.28, P = 2.75×10^-7^, OR = 0.65). Together the association signals from *LBX1* explain 1.4% of phenotypic variance. Our results identify two clinically relevant haplotypes in the *LBX1*-region with opposite effects on AIS risk. The study demonstrates the utility of haplotypes over un-phased SNPs for individualized risk assessment by more strongly delineating individuals at risk for AIS without compromising the effect size.

## Introduction

Adolescent idiopathic scoliosis (AIS) is a structural, lateral curvature of the spine greater than 10 degrees, without a known cause, diagnosed between age 10 and skeletal maturity. It affects 2–4% of the teenage population. Although scoliosis can be a primary or secondary feature in several dozen syndromes and can arise secondary to a variety of neuromuscular conditions, idiopathic scoliosis arising during puberty accounts for 80% of pediatric scoliosis. About 10–15% of youth diagnosed with AIS have progressive disease, and 2–4% progress to severe scoliosis [1]. Girls are much more likely than boys to have their spines progress to a severe degree [2]. AIS spinal curve is most likely to worsen during the adolescent growth spurt, sometimes leading to cosmetic deformity, back pain, psychological and social impairments, and respiratory and pulmonary limitations [3–5]. Usually an adolescent is first suspected of having AIS either during pediatric physical examination or during school screening [6].

The genetic component of AIS is both clear and significant as previously described [7–9], with heritability reported between 0.38 [10] and 0.87 [11]. Several genome-wide association studies (GWAS) have established a strong link to *LBX1* (Ladybird homeobox 1) [12–15], and a recent meta-study has confirmed *LBX1* as a major susceptibility locus for AIS in both Asian and Caucasian populations [16]. *LBX1* is a homeobox transcription factor. Its involvement in spinal cord differentiation and patterning, and somatosensory signal transduction make it a strong biological candidate for AIS [17, 18]. In addition to *LBX1* three other loci; *GPR126*, *SOX9* and *CHL1*, are putatively associated with AIS [14, 19]. The primary objective of the present study is to use GWAS to further resolve the genetic components of *LBX1* in AIS and to enhance individualized risk assessment in a Caucasian population.

## Subjects and Methods

### Study Subjects

Quorum Review IRB (Seattle, WA 98101) reviewed and approved the study (Quorum file # 23147/1). Adult subjects and controls provided written informed consent in accordance with study protocols. For minors, both an assent form (signed by the child) and a parental consent form were provided. Patients diagnosed with scoliosis were recruited with assistance from collaborating spine surgeons across the United States and control subjects were recruited from the general population. All data was de-identified.

### Inclusion Criteria

Female subjects were included if they had been diagnosed with idiopathic scoliosis between the ages of 10 and 13 and had reached skeletal maturity. Skeletal maturity was defined by a Risser score of 5 or by chronological age (≥16 years or 2 years post menarche). Risser scores quantify the ossification of the iliac apophysis on an anterior-posterior radiograph of the pelvis) [20]. Idiopathic scoliosis has been historically classified as “juvenile” if diagnosed between the ages of 3 and 10 and as “adolescent” if diagnosed from age 10 up until skeletal maturity [21]. Patients with congenital, juvenile, or adult-onset scoliosis, and patients diagnosed with syndromic scoliosis, spine trauma, myopathy, cerebral palsy, or other neurologic condition, were excluded.

### Phenotyping and Medical Record Review

All clinical data were collected and analyzed prior to genotyping. Medical records and spinal radiographs of each patient were blinded and reviewed by an orthopedic surgeon (JWO) who assessed Cobb angle measurements, Risser scores, and Lenke classification to provide a diagnosis [22]. Progression to a severe phenotype (curve) was defined according to usual clinical criteria; progression to a >40° curve in an adolescent, or progression to a >50° curve in an adult [23]. Mild and moderate AIS phenotypes were defined by Cobb angles of 10–24° and 25–40° respectively.

### DNA Extraction

DNA was obtained from saliva samples, collected using the Oragene DNA Self-Collection Kit (OG-300 Tube Format) (DNA Genotek; Ottawa, Ontario, Canada). DNA was extracted using an automated instrument, AutoPure LS (Qiagen; Valencia, CA), and manufacturer's reagents and protocols. DNA quality was evaluated by calculating absorbance ratio OD_260_/OD_280_, and DNA quantification was measured using Picogreen reagent (Invitrogen; Carlsbad, CA).

### Genotyping

Genotyping was performed on AIS cases and controls using the Affymetrix HuSNP 6.0 Microarray, with 906,600 SNPs (Affymetrix; Santa Clara, CA). Microarray processing was completed per manufacturer’s protocols. Genotypes were extracted from the scanned images using the Birdseed version 2 algorithm of the Affymetrix Power Tools package (v 1.10.2).

### Sample Quality Control

Samples were excluded from the analysis if they failed any of the following quality thresholds: (a) an individual was a relative of another subject in the study ($\hat{\pi }$ ≥ 0.20) using the Identity By State (IBS) estimation implemented in PLINK; (b) Affymetrix Quality Control (QC) call rate of < 95%; (c) gender ambiguity or error, determined using the observed genotypes of SNPs on chromosomes X and Y.

### SNP Quality Control

SNPs that failed any of the following quality metrics were excluded from the study: (a) p ≤10^–3^ in a test for deviation from Hardy-Weinberg Equilibrium (HWE) in cases and controls; (b) Minor allele frequency ≤3%; (c) SNP call rate ≤98%; and (d) visual inspection of genotype clusters for the 500 most strongly associated SNPs. SNPs from copy number variant regions and SNPs known to have frequent Mendelian or genotyping errors in other studies [24] were also removed.

### SNP Association Testing

After all quality control measures were performed, genetic association was determined using the whole-genome association analysis toolset, PLINK (v 1.07) [25]. Differences in allele frequencies between AIS case and control subjects were tested for each SNP using Cochran-Armitage Trend test. The allelic odds ratios were calculated along with 95% confidence interval. SNPs that passed the quality control parameters were used to generate Quantile-Quantile (QQ) plots ranking the observed χ^2^ values and plotting them against their expected values. Principal component analysis (PCA) was performed using EIGENSTRAT [26]. Haplotype-based association tests were calculated by 1-degree of freedom χ^2^ test, along with their respective odds ratios using R [27]. Different inheritance models were analyzed using logistic regression, which was also used to test for independence of SNP/haplotype effects and to calculate the phenotypic variance. The variance explained by a logistic regression model was calculated using the Cox Snell and Nagelkerke pseudo R2 method which is similar to the R2 concept of linear regression [28].

### Principal Component Analysis (PCA)

Validation of self-reported ethnicity was performed by PCA with the EIGENSTRAT program. To account for linkage disequilibrium (LD) effects, we performed LD pruning using a window size of 50 SNPs, window increment of 5 SNPs and LD threshold of r^2^>0.2. We performed PCA on the set of LD pruned markers and analyzed the top 10 eigenvectors. We then eliminated self-reported Caucasian samples that fell outside of the core European cluster. Subsequently we performed a secondary PCA on the core European cluster to determine any remaining population substructure.

### Imputation Analysis

IMPUTE2 (ver.2.1.2) [29] was used for imputing SNPs against the 1000-genomes (phase 1 data, v3). We performed the pre-phasing step on the genotyped samples prior to imputing haplotypes using a set of reference panel haplotypes from 1000 genomes. Imputation of the targeted region was carried out within ±2.5 Mb of the main marker of interest. Only SNPs that passed the confidence score of ≥0.9 and call rate of 0.98 with MAF>0.03 from imputation were retained for further analysis. The resulting haplotypes were used for haplotype based association tests.

## Results

An initial cohort of 906 AIS cases and 1480 controls who self-reported being of European descent was genotyped using Affymetrix HuSNP 6.0 Microarray chip. After applying the sample quality filters and subsequently eliminating samples of admixture using PCA (Fig. A in S2 File) a final population of 620 AIS case and 1,287 control samples remained for further analysis. Since the case-population was recruited from surgical practices, the subjects tended to have more severe scoliosis; the 620 cases included 495 with severe AIS, 63 with moderate and 62 with mild AIS. After applying the SNP quality filters 457,206 SNPs remained for analysis. The genomic inflation factor (λ) was estimated to be λ = 1.08 as shown by QQ plot in Fig. B in S2 File.

After visual inspection of the genotype clusters of the top 500 associated SNPs, we identified rs11190878 (P_trend_ = 4.18×10^–9^, OR = 0.63) in the *LBX1* locus as associated with AIS (Fig. C in S2 File). None of the other genotyped markers reached the genome-wide significance threshold (P<5×10^–8^). Results for the 30 most strongly AIS-associated SNPs from the GWAS are shown in Table A in S2 File.

**Fig 1:**
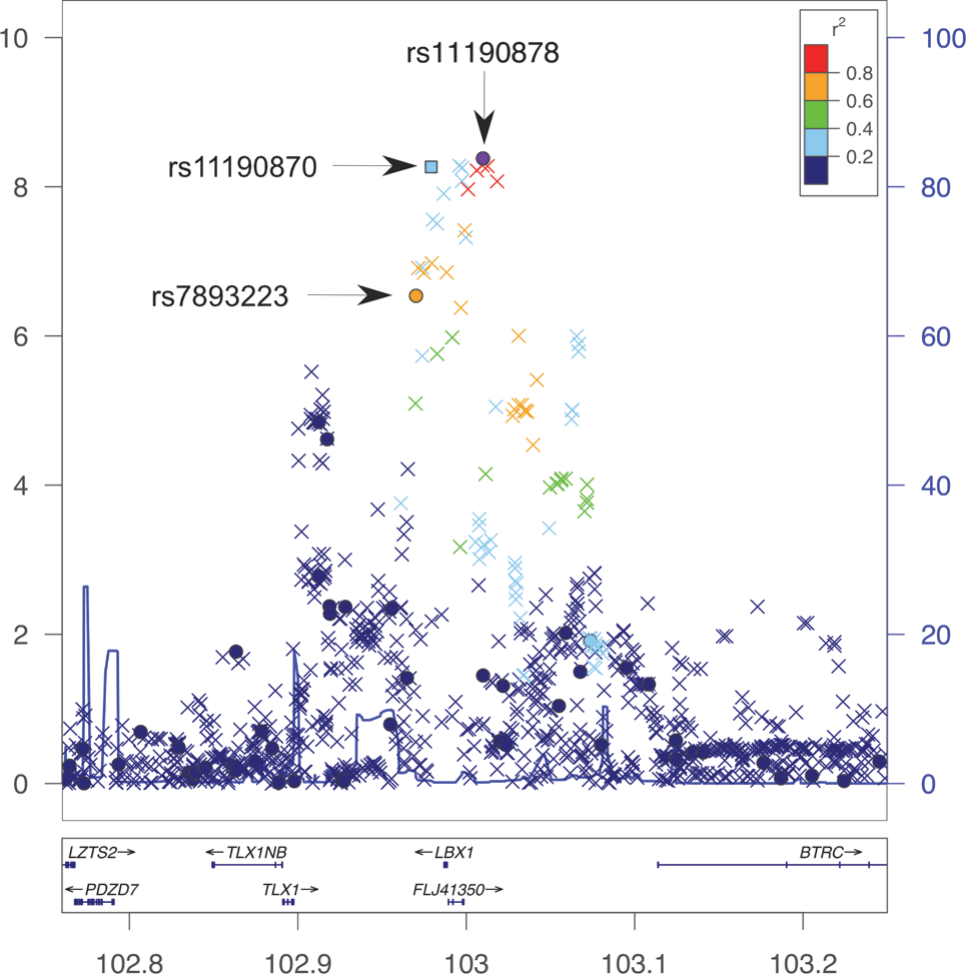
Regional Association Plot of *LBX1*. The panel shows-log10 of p-values for genotyped SNPs (●) and imputed SNPs (×) plotted against Chromosomal positions (hg19) in MB along the X-axis and-log10(P_trend_) values along the Y-axis. The European genetic recombination rate estimated from 1000G data is shown in blue line. Key SNPs are identified by arrows. The SNP rs11190870 that originally was reported to be associated with AIS was imputed in the present dataset. The gene identified as *FLJ41350* immediately upstream of *LBX1*, has recently been characterized as *LBX1-AS1* (LBX1 antisense RNA 1).

Through imputation of the *LBX1* region we identified 10 additional markers that passed the threshold of P_trend_ = 5×10^–7^ as shown in Table 1 (excluding markers in perfect LD). The table include rs11190870 (P_imputed_ = 5.43×10^–9^, OR = 0.66), previously reported to be associated with AIS is Asian populations, with association strength similar to rs11190878 identified here in a Caucasian population. The association plot of genotyped and imputed markers in the *LBX1* region (Fig. 1) indicates that rs11190878 is the most strongly associated marker in this region. The marker rs11190870 originally reported in Asian population, is in modest LD with rs11190878 (r^2^ = 0.40, D' = 0.81). To investigate if the association signals observed with rs11190870 and rs11190878 are independent, we selected the 2 strongly associated genotyped markers from this study (rs7893223 and rs11190879) together with the imputed marker rs11190870 for haplotype analysis. We found six distinct haplotypes (frequencies > 0.01) at the *LBX1* locus as shown in Table 2. The table shows two significantly associated haplotypes from the *LBX1* locus (hap-2 and hap-6) with opposite effects. To investigate the inheritance pattern for the risk (hap-6) and protective (hap-2) haplotype we evaluated the effect size of the haplotype-pairs on AIS (Table 3). It is evident that the risk haplotype (TTA) only is associated with AIS under a recessive inheritance model, whereas the protective haplotype (CCG) appears to have an additive effect. To confirm which genetic inheritance model best explains the protective haplotype hap-2 (CCG) we applied logistic regression using the most common haplotype hap-6 (TTA) as reference. These results (Table 4) show the co-dominant model as having the lowest AIC indicating that this model best explains the inheritance pattern of the protective haplotype (P = 2.09×10^–15^). We also examined the inheritance models for the remaining 4 haplotypes together (hap-1, hap-3, hap-4 and hap-5) again using hap-6 (TTA) as reference. Contrary to hap-2, this group only has minimal effect (Table B in S2 File), but similar to hap-2 this analysis also supports a recessive inheritance model for hap-6. The variance explained by *LBX1* was estimated using a multivariate logistic regression that included the haplotypes with the inheritance models established above. This analysis, summarized in Table C in S2 File, indicates that the risk factors have independent effects and together explain 1.4% of the variance for AIS.

**Table 1:** Adolescent Idiopathic Scoliosis (AIS) show Genetic Association at LBX1 in a Caucasian Population.

SNP	Variant Location	Gene	Position	Minor allele	Case _freq_ (n = 620)	Control _freq_ (n = 1287)	P _*trend*_	OR [CI 95%]
rs7893223*	intergenic	TLX1—LBX1	102,970,161	C	0.233	0.312	2.90×10^–7^	0.67[0.57–0.78]
rs1535462	intergenic	TLX1—LBX1	102,973,872	G	0.386	0.477	1.22×10^–7^	0.69[0.60–0.79]
rs11190870^#^	intergenic	TLX1—LBX1	102,979,207	C	0.349	0.449	5.43×10^–9^	0.66[0.57–0.76]
rs35999315	intergenic	TLX1—LBX1	102,979,555	T	0.226	0.308	1.06×10^–7^	0.66[0.56–0.77]
rs1322331	downstream	LBX1	102,986,589	C	0.384	0.481	1.24×10^–8^	0.67[0.58–0.77]
rs594791	ncRNA_intronic	LBX1-AS1	102,995,796	T	0.342	0.441	5.23×10^–9^	0.66[0.57–0.76]
rs678741	ncRNA_intronic	LBX1-AS1	102,997,581	G	0.373	0.473	5.56×10^–9^	0.66[0.58–0.76]
rs12773591	downstream	LBX1-AS1	102,999,019	G	0.214	0.297	3.83×10^–8^	0.64[0.55–0.75]
rs10883597	intergenic	LBX1-AS1—BTRC	102,999,754	T	0.334	0.426	4.79×10^–8^	0.68[0.59–0.78]
rs11593547	intergenic	LBX1-AS1—BTRC	103,001,055	T	0.222	0.310	1.08×10^–8^	0.63[0.54–0.74]
rs11190878*	intergenic	LBX1-AS1—BTRC	103,009,908	G	0.237	0.329	4.18×10^–9^	0.63[0.54–0.74]
rs17686462	intergenic	LBX1-AS1—BTRC	103,018,321	T	0.241	0.331	8.47×10^–9^	0.64[0.55–0.75]

The 12 SNPs included in the table were found to be strongly association with AIS (P_trend_<5×10–7). The SNPs span a 48kb region around LBX1 on chromosome 10q24.31 which also include LBX1-AS1; a non-coding anti-sense RNA expected to have important post-transcriptional regulatory properties. Fig. 1 show a total of 22 markers from this region with P_trend_<5×10–7, however, markers in perfect LD have been removed from the table. Genotype clusters for rs11190878 and rs7893223 are shown in Fig. C in S2 File. # Indicates the SNP previously reported as the marker most strongly associated with AIS.

* Indicates SNPs that were genotyped in the present GWAS. All other SNPs were imputed using a reference panel of haplotypes from 1000 genomes.

**Table 2:** LBX1 Haplotypes Associated with Adolescent Idiopathic Scoliosis.

SNP	hap-1	hap-2	hap-3	hap-4	hap-5	hap-6
rs7893223	C	C	T	T	T	T
rs11190870*	C	C	C	C	T	T
rs11190878	A	G	A	G	G	A
						
Case _frequency_	0.028	0.202	0.111	0.008	0.028	0.622
Control _frequency_	0.030	0.280	0.122	0.019	0.032	0.518
P _chisq_	0.749	2.75×10^–7^	0.354	0.013	0.465	1.25×10^–9^
OR	0.93	0.65	0.90	0.36	0.86	1.56

Two strongly associated haplotypes exists at the LBX1 locus. Haplotype 6 show stronger risk association than any of the individual SNPs in the region suggesting that the risk allele is not one of the SNPs presently reported. Surprisingly, a second haplotype (haplotype 2) shows strong protective effect. This suggests that multiple variants with different risk profiles exist at the *LBX1* locus. The six haplotypes labeled hap-1 to hap-6 are shown vertically.

* SNP rs11190870 was imputed.

**Table 3:** LBX1 Haplotype Pairs Associated with AIS.

Haplotype 1	Haplotype 2	Cases (frequency)	Controls (frequency)	OR [Cl 95%]	χ2 P_value_
TTA	TTA	227 (0.366)	336 (0.261)	1.63[1.33–2.07]	2.46×10^–6^
TTA	otherhap*	142 (0.229)	281 (0.218)	1.06[0.84–1.34]	0.59
TTA	CCG	171 (0.276)	375 (0.291)	0.93[0.75–1.15]	0.48
CCG	otherhap*	45 (0.073)	145 (0.113)	0.62[0.43–0.87]	6.10×10^–3^
CCG	CCG	17 (0.027)	99 (0.077)	0.34[0.20–0.57]	2.26×10^–5^
otherhap*	otherhap*	18 (0.029)	51 (0.040)	0.72[0.42–1.25]	0.24

The table reflects the number of cases and controls observed with specific haplotype-pairs.

* otherhap denotes all the other haplotypes other than TTA and CCG.

**Table 4:** Genetic Models underlying AIS at LBX1 Locus Indicate a Codominant Protective Factor Residing on the CCG Haploytype as Calculated using the Ancestral Haplotype TTA as the Reference Allele.

Model	Haplotype	Control	Case	OR	AIC	P-Value
Codominant	TTA/TTA	227 (0.55)	336 (0.41)	1	1540.7	2.09×10^–15^
	TTA/CCG	171 (0.41)	375 (0.46)	0.68		
	CCG/CCG	17 (0.04)	99 (0.12)	0.25		
Dominant	TTA/TTA	227 (0.55)	336 (0.41)	1	1553.3	5.76×10^–10^
	TTA/CCG-CCG/CCG	188 (0.45)	474 (0.59)	0.59		
Recessive	TTA/TTA-TTA/CCG	398 (0.96)	711 (0.88)	1	1548.5	4.26×10^–12^
	CCG/CCG	17 (0.04)	99 (0.12)	0.31		
Overdominant	TTA/TTA-CCG/CCG	244 (0.59)	435 (0.54)	1	1569.7	1.80×10^–2^
	TTA/CCG	171 (0.41)	375 (0.46)	0.81		
Additive	—	—	—	0.59	1542	5.71×10^–15^

Values listed under Case and Control indicates the observed individual counts from the present dataset with percentages shown in brackets. AIC denotes Akaike Information Criterion derived from Logistic Regression analysis. P-Value is calculated using log likelihood ratio test and OR denotes Odds Ratio.

A comparison of results from the present study to previously reported GWAS associations is shown in Table 5. The table shows strong replication of rs11190870 in *LBX1* and nominal support for rs6570507 in GPR126 [30]. The present data does not support association to rs10510181 in CHL1 [14] or rs12946942 in SOX9 [19] in our Caucasian population. A lack of association between *CHL1* and AIS has also recently been reported in a Han Chinese population [31].

**Table 5:** Comparison of Previously Reported Associations to AIS with Results from the Present Study.

						Previously Reported Data					This Study			
SNP	Gene	Chr	Position	MA		Case _freq_	CTL _freq_	P _trend_	OR		Case _freq_	CTL _freq_	P _trend_	OR
rs10510181	CHL1	3	191,047	A		0.370	0.320	8.22×10^–7^	1.29		0.328	0.336	0.55	0.96
rs6570507	GPR126	6	142,679,572	A		0.499	0.430	3.78×10^–8^	1.32		0.319	0.290	0.04	1.15
rs11190870	LBX1	10	102,979,207	C		0.325	0.435	2.80×10^–18^	0.63		0.349	0.449	5.43×10^–9^	0.66
rs12946942	SOX9	17	69,236,998	T		0.258	0.211	9.58×10^–6^	1.30		0.096	0.083	0.13	1.18

The marker rs11190870 at *LBX1* is significantly associated in our European population while rs6570507 at *GPR126* is nominally associated in the present study. Our study does not show evidence of association to AIS in *CHL1* and *SOX9*. The marker rs10510181 at *CHL1* was originally identified in a European population while markers at *GPR126*, *LBX1* and *SOX9* were discovered in Asian populations. rs10510181 and rs11190870 were imputed in this study. Previously Published Data reported from [14, 19, 30].

## Discussion

This study validates the association of *LBX1* with AIS in subjects of European descendence and presents evidence of two independent, opposing association signals at the *LBX1* locus. Our results show rs11190870 and rs11190878 with equally strong association to AIS, yet the two markers are only in moderate LD with one another (r^2^ = 0.40, D' = 0.81). This raises the question whether their respective association signals are independent or share an ancestral origin. Based on our haplotype analysis, it is evident that the ancestral (common) alleles of both rs11190870 (T, freq = 0.551) and rs11190878 (A, freq = 0.671) reside on the ancestral haplotype hap-6 (TTA) (Table 2). It is therefore reasonable to conclude that the Asian and European populations share the ancestral haplotype associated with a sharply increased risk for AIS.

Hap-6 (TTA, P = 1.25×10^–9^, OR = 1.56), the most common of the six haplotypes, is associated with an increased risk for AIS (Table 2). Our results also reveal a second haplotype, hap-2 (CCG, P = 2.75×10^–7^, OR = 0.65), with a protective effect. Further investigation of the inheritance patterns of the risk (hap-6) and protective (hap-2) haplotypes demonstrated that hap-6 (TTA) is inherited in a recessive fashion, whereas hap-2 (CCG) is inherited in a co-dominant fashion in the Caucasian population (Tables 3 and 4). Individuals carrying both hap-2 (CCG) and hap-6 (TTA) have neither increased nor decreased risk, suggesting that these two haplotypes neutralize each other. It remains to be determined if a protective haplotype also exists in Asian populations.

The power of haplotypes as clinical predictors compared to un-phased SNPs become clear in Tables 1–3, which in the case of rs11190878, show that the case frequency of the two homozygous genotypes (nnA/nnA + nnG/nnG) is 0.616, whereas the corresponding frequency of the two associated haplotypes (TTA/TTA + CCG/CCG) is 0.394. This comparison demonstrates the utility of haplotypes over un-phased SNPs by more strongly delineating the group of individuals at risk for AIS without compromising the effect size. We also estimate the phenotypic variance explained by hap-2 and hap-6 at *LBX1* for AIS to be 1.4% (Table C in S2 File), which is large for a single locus in a complex disease.

In Fig. D in S2 File we show the frequency of patients grouped by disease severity and by haplotypes. The graph shows a clear trend whereby the TTA-TTA group has an increased frequency of moderate and severe patients and reduced frequency of mild patients, while patients with CCG-other and CCG-CCG has a reduced frequency of severe patients and increased frequency of mild patients. The trend fails to reach statistically significance in our study and it will be interesting to see if the correlation between haplotypes and severity can be independently confirmed.

The genomic architecture of the *LBX1* locus (Fig. E in S2 File) reveals a highly conserved gene with extensive regulatory mechanisms. Of particular interest are the head-to-head orientation of *LBX1* with its antisense counterpart *LBX1-AS1*, because antisense transcription increasingly is being recognized as an important regulator of gene expression [32]. The figure also shows some very extensive CpG islands (shown in Green) that flank *LBX1*and are a hallmark for epigenetic transcriptional regulation. Indeed, it has been shown that antisense expression can affect methylation patterns and lead to long-term repression [33]. Finally, Fig. E shows a pattern of remarkably extensive nucleotide conservation across vertebrate-species that not only include the protein coding regions but extends about 10 kb down-stream of *LBX1*. Together, the presence of *LBX1-AS1*, the CpG islands and the conserved regions suggests that *LBX1* expression is very tightly regulated. A detailed functional description of the regulatory mechanisms that govern the *LBX1* locus is not yet available, but we expect that sequencing of the *LBX1* locus will identify rare and private mutations with large effects to segregate in some AIS families.

## Supporting Information

S1 File Includes all genotypes and case status information necessary to reproduce the imputation and haplotype analysis reported here. (TXT)

S2 File Includes the following files: Fig. A. PCA Plots. PCA plots of the 2 first eigenvectors at different geographical resolution are shown. Panel A show the Case and Control samples that self-reported as being of European ancestry (blue and green circles) together with previously characterized hapmap samples of African, Asian and European origin (black, purple and red circles). Samples determined by PCA to be admixed (blue circles) were eliminated from the study. A second PCA was performed on samples of PCA-verified European ancestry (blue rectangle) and shown in Panel B. In Panel B the cases are represented in green and controls in red. As suggested in panel B the genetic variance between case and control samples is minimal and the genomic inflation measure lambda λ = 1.08. **Fig. B**. Quantile-Quantile (Q-Q) plot. QQ plot of the allelic association analysis of expected versus observed χ2. The genomic inflation factor, λ = 1.08, indicate minimal stratification that does not require further PCA-adjustment. The QQ-plot was generated using 77,366 SNPs in linkage equilibrium (r^2^<0.2). **Fig. C**. Genotype Cluster Plots. Genotype cluster plots for the two genotyped SNPs in Table 1 are shown here using the A and B allelic intensity values. The distinct colors for each of the homozygous and heterozygous clusters represent cases (n = 853) and controls (n = 1,368). **Fig. D**. Haplotype vs. Severity Bar Plot. The bar-plot shows the case percentages for each of the three AIS classes (mild, moderate and severe) per haplotype groups. The graph shows a clear trend whereby the TTA-TTA group has an increased frequency of severe patients and reduced frequency of mild patients, while patients with CCG-other and CCG-CCG has a reduced frequency of severe patients and increased frequency of mild patients. Severity among individuals with TTA-CCG show equal frequency across the three groups, which supports the notion that the effects of TTA and CCG neutralize each other. **Fig. E**. Genomic architecture of the *LBX1* locus. The genomic architecture around *LBX1* on chromosome 10q24.31 reveals a highly conserved gene with extensive regulatory mechanisms. Of particular interest are the head-to-head orientation of *LBX1* with is antisense counterpart *LBX1-AS1* together with the very extensive CpG islands (shown in Green) that include and flank *LBX1*. In addition to the highly conserved coding regions of *LBX1* a series of conserved regions are shown in blue mostly downstream of *LBX1*. **Table A**. Top 30 SNPs from GWAS. **Table B**. Logistic Regression Analysis of the Four Minor Haplotypes Combined. **Table C**. Multivariate Conditional Logistic Regression.
(DOCX)
