# Supplementary material for: Haplotypes at LBX1 Have Distinct Inheritance Patterns with Opposite Effects in Adolescent Idiopathic Scoliosis
Source: PLoS One. 2015 Feb 12;10(2):e0117708. doi: 10.1371/journal.pone.0117708 (PMC4326419; doi:10.1371/journal.pone.0117708)
Supplement: S2 File — PCA Plots. PCA plots of the 2 first eigenvectors at different geographical resolution are shown. Panel A show the Case and Control samples that self-reported as being of European ancestry (blue and green circles) together with previously characterized hapmap samples of African, Asian and European origin (black, purple and red circles). Samples determined by PCA to be admixed (blue circles) were eliminated from the study. A second PCA was performed on samples of PCA-verified European ancestry (blue rectangle) and shown in Panel B. In Panel B the cases are represented in green and controls in red. As suggested in panel B the genetic variance between case and control samples is minimal and the genomic inflation measure lambda λ = 1.08. Fig. B. Quantile-Quantile (Q-Q) plot. QQ plot of the allelic association analysis of expected versus observed χ2. The genomic inflation factor, λ = 1.08, indicate minimal stratification that does not require further PCA-adjustment. The QQ-plot was generated using 77,366 SNPs in linkage equilibrium (r2<0.2). Fig. C. Genotype Cluster Plots. Genotype cluster plots for the two genotyped SNPs in Table 1 are shown here using the A and B allelic intensity values. The distinct colors for each of the homozygous and heterozygous clusters represent cases (n = 853) and controls (n = 1,368). Fig. D. Haplotype vs. Severity Bar Plot. The bar-plot shows the case percentages for each of the three AIS classes (mild, moderate and severe) per haplotype groups. The graph shows a clear trend whereby the TTA-TTA group has an increased frequency of severe patients and reduced frequency of mild patients, while patients with CCG-other and CCG-CCG has a reduced frequency of severe patients and increased frequency of mild patients. Severity among individuals with TTA-CCG show equal frequency across the three groups, which supports the notion that the effects of TTA and CCG neutralize each other. Fig. E. Genomic architecture of the LBX1 locus. The genomic architectu [file pone.0117708.s002.docx]

**S2 File.docx**

Supplementary Figures and Tables

**Opposing Haplotypes at the LBX1 Locus Affects Risk for Adolescent Idiopathic Scoliosis.**

**.**

Rakesh Chettier, Lesa Nelson, James W. Ogilvie, Hans M. Albertsen and Kenneth Ward


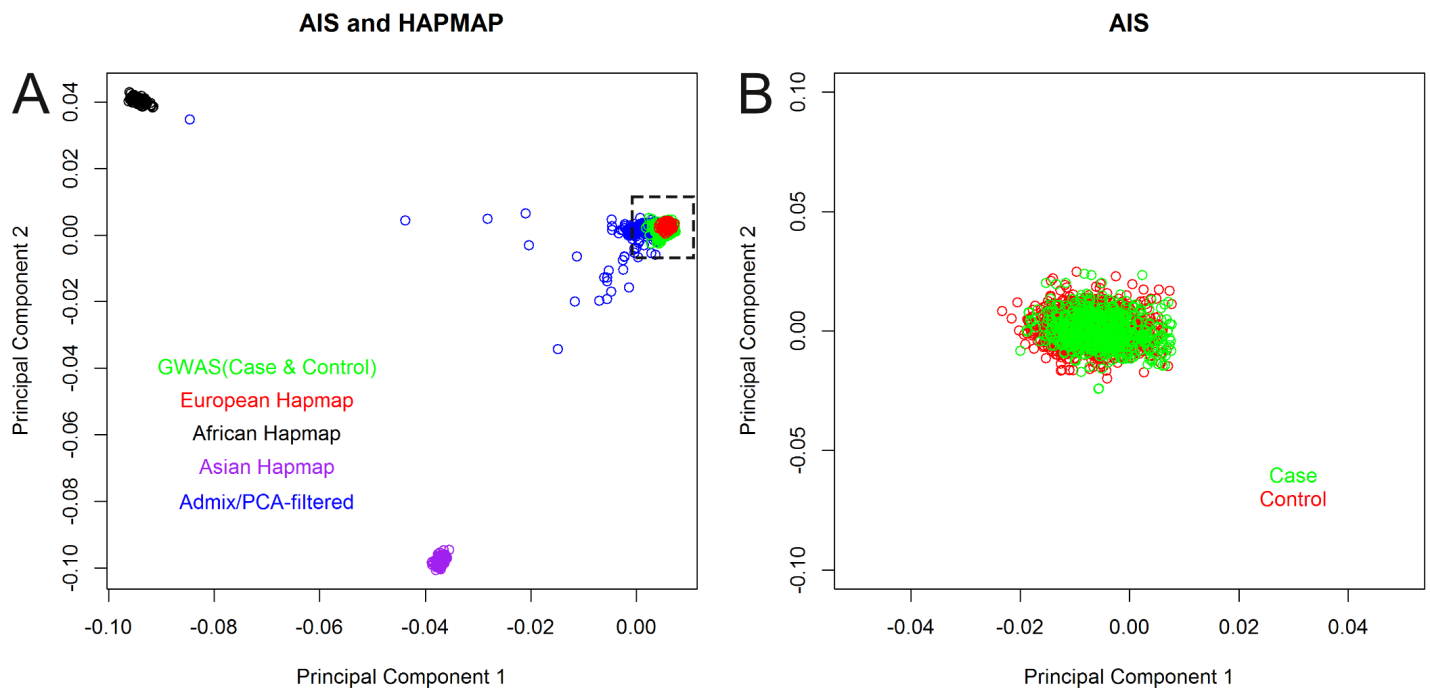


**Figure A. PCA Plots.** PCA plots of the 2 first eigenvectors at different geographical resolution are shown. Panel A show the Case and Control samples that self-reported as being of European ancestry (blue and green circles) together with previously characterized hapmap samples of African, Asian and European origin (black, purple and red circles). Samples determined by PCA to be admixed (blue circles) were eliminated from the study. A second PCA was performed on samples of PCA-verified European ancestry (blue rectangle) and shown in Panel B. In Panel B the cases are represented in green and controls in red. As suggested in panel B the genetic variance between case and control samples is minimal and the genomic inflation measure lambda λ=1.08.


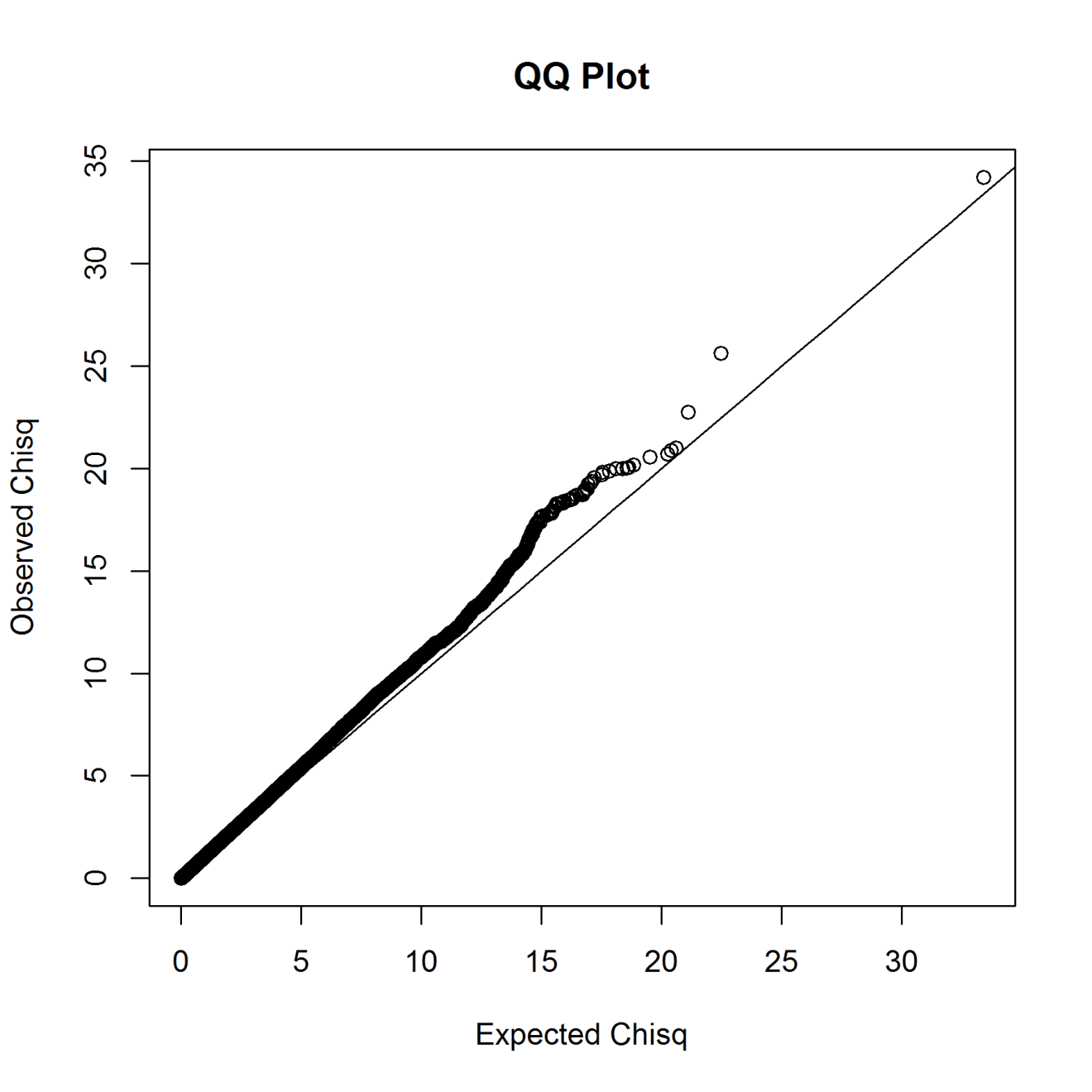


**Figure B.** **Quantile-Quantile (Q-Q) plot.** QQ plot of the allelic association analysis of expected versus observed χ^2^. The genomic inflation factor, λ=1.08, indicate minimal stratification that does not require any further PCA- based adjustment. The QQ-plot was generated using 454,050 SNPs.

**
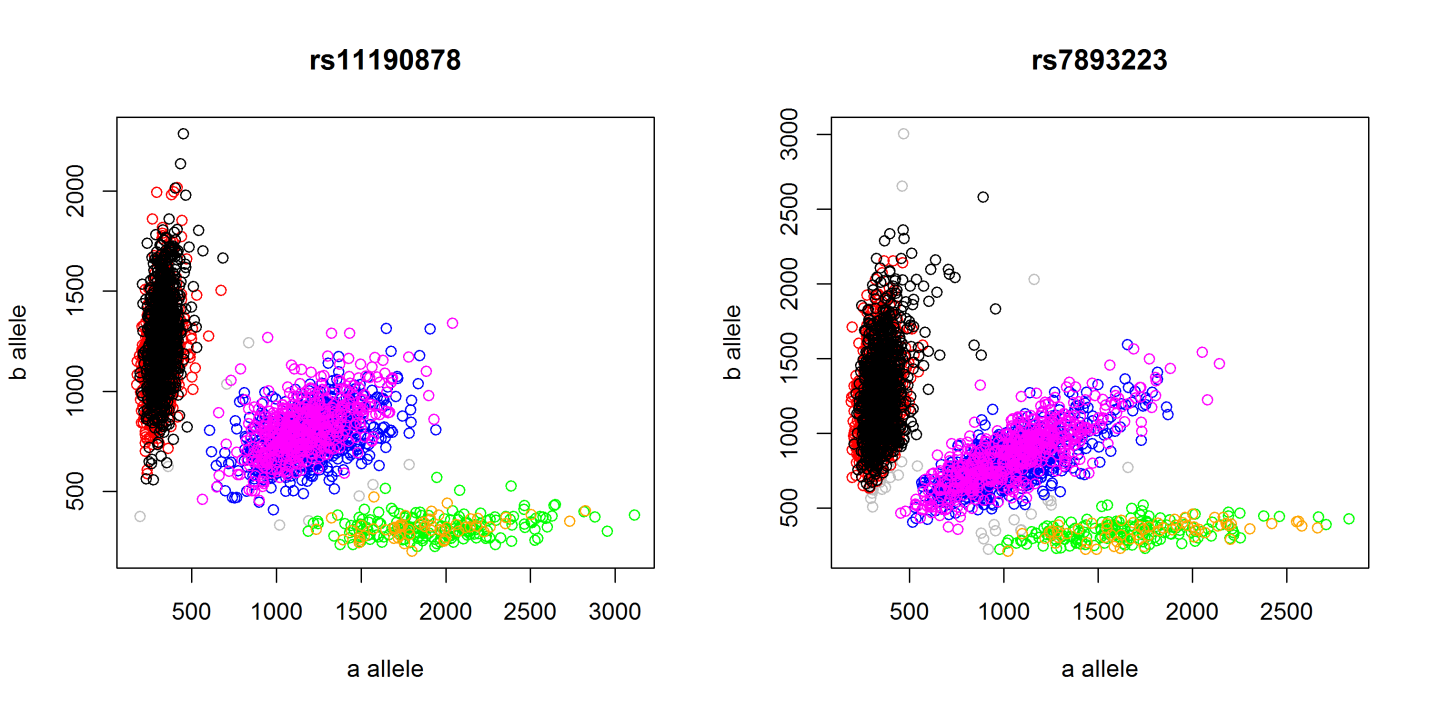
**

**Figure C. Genotype Cluster Plots.** Genotype cluster plots of the two SNPs present in Table 1 are shown here using the A and B allelic intensity values. The distinct colors for each of the homozgyous and heterozygous clusters represent all samples from this study including cases (n=853) and controls (n=1,368).


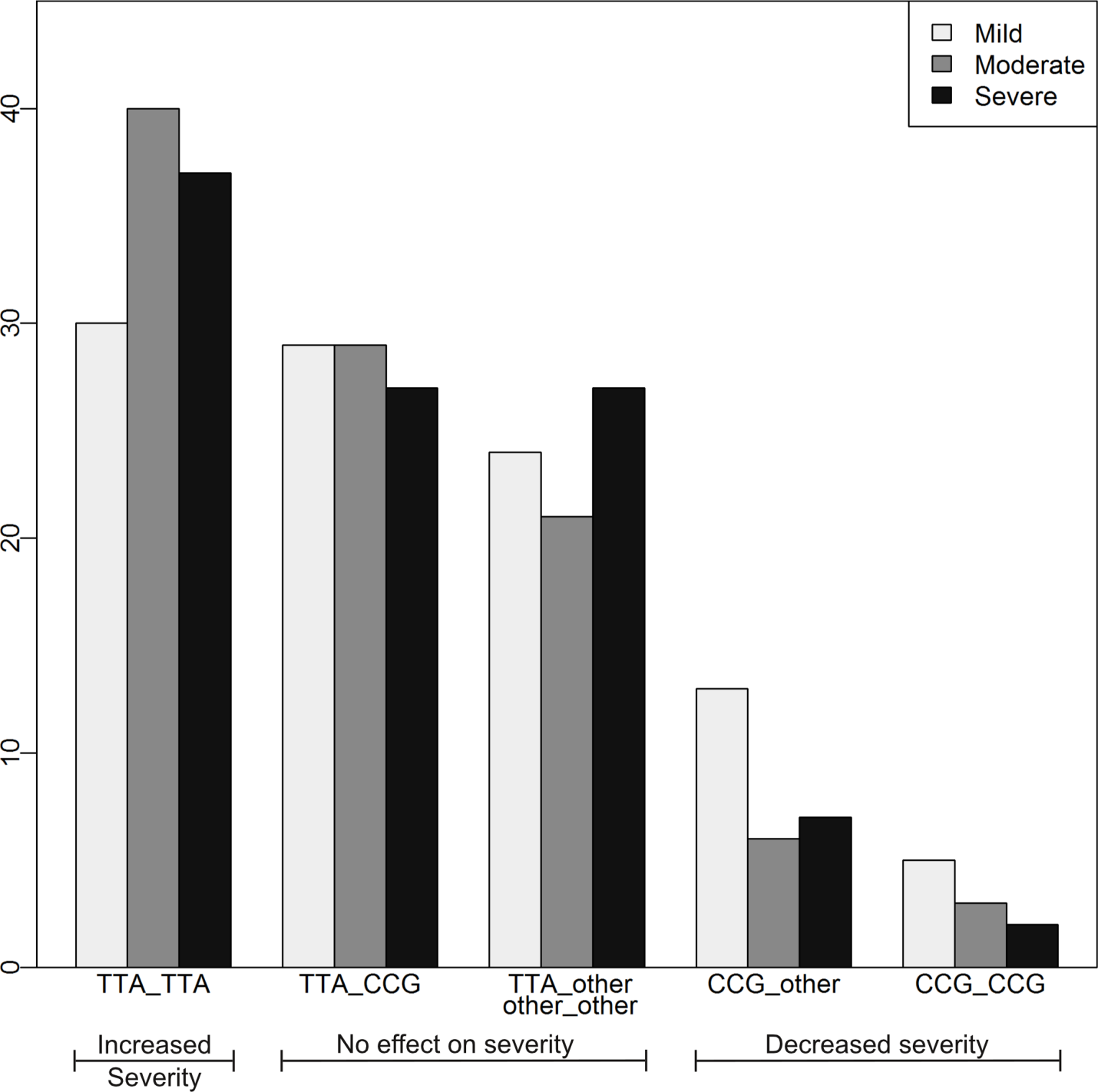


**Figure D. Haplotype vs. Severity Bar Plot.** The bar-plot shows the case percentages for each of the three AIS classes (mild, moderate and severe) per haplotype groups. The graph shows a clear trend whereby the TTA-TTA group has an increased frequency of severe patients and reduced frequency of mild patients, while patients with CCG-other and CCG-CCG has a reduced frequency of severe patients and increased frequency of mild patients. Severity among individuals with TTA-CCG show equal frequency across the three groups, which supports the notion that the effects of TTA and CCG neutralize each other.


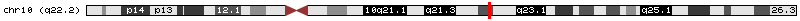


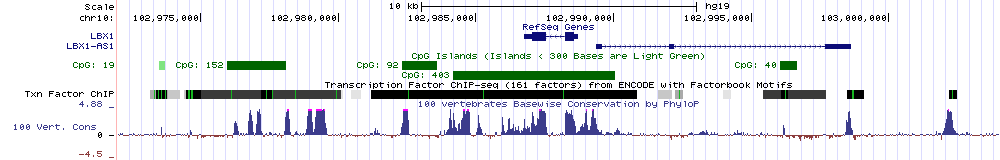


**Figure E.** **Genomic architecture of the *LBX1* locus.** The genomic architecture around *LBX1* on chromosome 10q24.31 reveals a highly conserved gene with extensive regulatory mechanisms. Of particular interest are the head-to-head orientation of *LBX1* with is antisense counterpart *LBX1-AS1* together with the very extensive CpG islands (shown in Green) that include and flank *LBX1*. In addition to the highly conserved coding regions of *LBX1* a series of conserved regions are shown in blue mostly downstream of *LBX1*.

| **Table A** Top 30 AIS associated SNPs from this GWAS | | | | | | | | | | | |
| --- | --- | --- | --- | --- | --- | --- | --- | --- | --- | --- | --- |
| **SNP** | **Chr** | **Base-pair** | **A1** | **A2** | **Case_freq_** | **Control_freq_** | **P _trend_** | **OR** | **Cl (l95-u95)** | **Location** | **Gene** |
| rs2209158 | 1 | 110,795,018 | C | A | 0.458 | 0.383 | 8.59E-06 | 1.36 | 1.19-1.56 | intergenic | KCNC4(dist=18344),LOC440600(dist=33981) |
| rs10911810 | 1 | 186,036,398 | G | A | 0.543 | 0.468 | 1.46E-05 | 1.35 | 1.18-1.55 | ncRNA_intronic | MIR548F1 |
| rs10911813 | 1 | 186,053,171 | G | A | 0.442 | 0.519 | 7.45E-06 | 0.73 | 0.64-0.84 | ncRNA_intronic | MIR548F1 |
| rs7527490 | 1 | 186,074,918 | T | C | 0.440 | 0.518 | 7.15E-06 | 0.73 | 0.64-0.84 | ncRNA_intronic | MIR548F1 |
| rs7555906 | 1 | 186,082,316 | G | C | 0.448 | 0.524 | 9.19E-06 | 0.74 | 0.64-0.84 | ncRNA_intronic | MIR548F1 |
| rs4233125 | 1 | 186,082,632 | C | T | 0.449 | 0.525 | 1.19E-05 | 0.74 | 0.64-0.85 | ncRNA_intronic | MIR548F1 |
| rs4675296 | 2 | 203,693,559 | G | C | 0.070 | 0.037 | 1.49E-05 | 1.95 | 1.44-2.63 | intronic | ICA1L |
| rs12616799 | 2 | 203,811,847 | A | G | 0.069 | 0.036 | 7.54E-06 | 1.99 | 1.47-2.69 | intronic | ALS2CR8 |
| rs7603031 | 2 | 203,860,866 | C | G | 0.067 | 0.036 | 2.26E-05 | 1.94 | 1.43-2.63 | intergenic | ALS2CR8(dist=9806),NBEAL1(dist=18736) |
| rs9855248 | 3 | 191,751,195 | A | G | 0.477 | 0.400 | 7.16E-06 | 1.37 | 1.19-1.57 | intergenic | PYDC2(dist=571950),FGF12(dist=105987) |
| rs2609081 | 5 | 2,286,287 | T | C | 0.185 | 0.251 | 6.64E-06 | 0.68 | 0.57-0.80 | intergenic | IRX4(dist=403407),IRX2(dist=459992) |
| rs9283782 | 5 | 82,900,921 | T | C | 0.304 | 0.376 | 1.49E-05 | 0.73 | 0.63-0.84 | intergenic | VCAN(dist=22799),HAPLN1(dist=33096) |
| rs10478203 | 5 | 114,248,387 | A | C | 0.291 | 0.226 | 8.04E-06 | 1.41 | 1.21-1.65 | intergenic | KCNN2(dist=416190),TRIM36(dist=212072) |
| rs10074889 | 5 | 149,031,582 | G | A | 0.532 | 0.454 | 8.09E-06 | 1.36 | 1.19-1.56 | intergenic | ARHGEF37(dist=17055),PPARGC1B(dist=78233) |
| rs9406022 | 6 | 7,328,580 | T | C | 0.307 | 0.379 | 1.31E-05 | 0.73 | 0.63-0.84 | intronic | CAGE1 |
| rs1159199 | 8 | 31,808,119 | C | G | 0.130 | 0.085 | 1.57E-05 | 1.61 | 1.30-2.00 | intronic | NRG1 |
| rs2066367 | 10 | 16,411,683 | T | C | 0.490 | 0.413 | 7.40E-06 | 1.37 | 1.19-1.57 | intergenic | FAM188A(dist=509164),PTER(dist=67284) |
| rs3847398 | 10 | 30,265,480 | C | T | 0.412 | 0.491 | 4.91E-06 | 0.73 | 0.63-0.84 | intergenic | SVIL(dist=240750),KIAA1462(dist=36249) |
| rs4749516 | 10 | 30,268,579 | T | C | 0.379 | 0.453 | 1.16E-05 | 0.74 | 0.64-0.85 | intergenic | SVIL(dist=243849),KIAA1462(dist=33150) |
| rs1571764 | 10 | 30,273,031 | C | T | 0.413 | 0.492 | 4.43E-06 | 0.73 | 0.63-0.83 | intergenic | SVIL(dist=248301),KIAA1462(dist=28698) |
| rs11813606 | 10 | 32,692,720 | A | T | 0.274 | 0.210 | 1.06E-05 | 1.42 | 1.21-1.66 | intergenic | EPC1(dist=56607),CCDC7(dist=42321) |
| rs7095821 | 10 | 102,912,514 | G | A | 0.436 | 0.363 | 1.58E-05 | 1.36 | 1.18-1.56 | intergenic | TLX1(dist=14968),LBX1(dist=74219) |
| rs7893223 | 10 | 102,970,161 | C | T | 0.231 | 0.311 | 2.88E-07 | 0.67 | 0.57-0.78 | intergenic | TLX1(dist=72615),LBX1(dist=16572) |
| rs11190878 | 10 | 103,009,908 | C | T | 0.237 | 0.330 | 3.80E-09 | 0.63 | 0.54-0.74 | intergenic | FLJ41350(dist=11292),BTRC(dist=103882) |
| rs4886114 | 13 | 59,634,447 | T | C | 0.429 | 0.348 | 1.25E-06 | 1.41 | 1.22-1.62 | intergenic | PCDH17(dist=1331382),DIAPH3(dist=605276) |
| rs11647694 | 16 | 26,208,742 | C | T | 0.376 | 0.302 | 2.98E-06 | 1.40 | 1.21-1.61 | intergenic | HS3ST4(dist=59733),C16orf82(dist=869477) |
| rs10406613 | 19 | 30,276,911 | A | G | 0.334 | 0.266 | 5.51E-06 | 1.39 | 1.20-1.61 | intergenic | C19orf12(dist=70215),CCNE1(dist=25990) |
| rs201928 | 20 | 17,524,903 | T | C | 0.353 | 0.428 | 1.25E-05 | 0.73 | 0.63-0.84 | intronic | BFSP1 |
| rs1010304 | 20 | 40,101,647 | G | A | 0.029 | 0.063 | 1.12E-05 | 0.44 | 0.30-0.64 | intronic | CHD6 |
| rs927142 | X | 90,305,713 | A | G | 0.333 | 0.263 | 1.42E-05 | 1.40 | 1.21-1.62 | intergenic | TGIF2LX(dist=1127831),PABPC5(dist=383884) |

| **Table B** Genetic models underlying AIS at *LBX1* locus does not indicate a significant association for non-CCG haploytypes as calculated using the ancestral haplotype TTA as the reference allele. | | | | | | |
| --- | --- | --- | --- | --- | --- | --- |
| Model | Haplotype | Control | Case | OR | AIC | P-value |
| Codominant | TTA/TTA | 227 (0.59) | 336 (0.5) | 1 | 1384.3 | 2.03×10^-4^ |
|  | TTA/other | 142 (0.37) | 281 (0.42) | 0.75 |  |  |
|  | other/other | 18 (0.05) | 51 (0.08) | 0.52 |  |  |
| Dominant | TTA/TTA | 227 (0.59) | 336 (0.5) | 1 | 1383.9 | 2.03×10^-4^ |
|  | TTA/other-other/other | 160 (0.41) | 332 (0.5) | 0.71 |  |  |
| Recessive | TTA/TTA-TTA/other | 369 (0.95) | 617 (0.92) | 1 | 1387 | 5.84×10^-3^ |
|  | other/other | 18 (0.05) | 51 (0.08) | 0.59 |  |  |
| Overdominant | TTA/TTA-other/other | 245 (0.63) | 387 (0.58) | 1 | 1387.8 | 1.43×10^-2^ |
|  | TTA/other | 142 (0.37) | 281 (0.42) | 0.8 |  |  |
| Additive | --- | --- | --- | 0.62 | 1384.9 | 5.92×10^-4^ |
|  |  |  |  |  |  |  |
| Relative to the values calculated for the CCG haplotype shown in Table 4 the "other" haplotypes reported here do not show significant association with AIS. Values listed under Case and Control indicates the observed individual counts from the present dataset with percentages shown in brackets. | | | | | | |
| "other" denotes haplotypes hap-1, hap-3, hap-4 and hap-5 as defined in Table 2 | | | | | | |
| AIC denotes Akaike Information Criterion derived from Logistic Regression analysis | | | | | | |
| P-value is calculated using log likelihood ratio test | | | | | | |
| OR denotes Odds Ratio | | | | | | |

| **Table C** Multivariate conditional logistic regression | | | | | | |
| --- | --- | --- | --- | --- | --- | --- |
| Genetic variables(loci) | Alleles Haplotypes | Genetic Model | Beta (estimated) | OR_adj_ | 95% CI | P Value |
| hap2 (LBX1) | CCG | additive | -0.323 | 0.72 | [0.66-0.80] | 1.24E-03 |
| hap6 (LBX1) | TTA | Recessive | 0.267 | 1.31 | [1.15-1.48] | 3.20E-06 |
| Table shows the estimates of a multivariate logistic regression model fitted with genetic variables in *LBX1*. All of the genetic variables shown in the table are independent risk factors (p<0.05) for AIS and together they explain 1.4% of the variance in AIS. | | | | | | |
| Beta: logistic regression parameter | | | | | | |
